# Comparative Incorporation of PNA into DNA Nanostructures

**DOI:** 10.3390/molecules200917645

**Published:** 2015-09-23

**Authors:** Ronnie O. Pedersen, Jing Kong, Catalina Achim, Thomas H. LaBean

**Affiliations:** 1 Department of Chemistry, Duke University, 124 Science Drive, Durham, NC 27708-0354, USA; E-Mail: r.o.pedersen@gmail.com; 2 Department of Chemistry, Carnegie Mellon University, Pittsburgh, PA 15213, USA; E-Mail: jingk@andrew.cmu.edu; 3 Department of Materials Science and Engineering, North Carolina State University, 911 Partners Way, Raleigh, NC 27695-7907, USA

**Keywords:** peptide nucleic acid, oligonucleotide conjugates, modified oligonucleotides, DNA nanobiotechnology, nanomaterials

## Abstract

DNA has shown great promise as a building material for self-assembling nanoscale structures. To further develop the potential of this technology, more methods are needed for functionalizing DNA-based nanostructures to increase their chemical diversity. Peptide nucleic acid (PNA) holds great promise for realizing this goal, as it conveniently allows for inclusion of both amino acids and peptides in nucleic acid-based structures. In this work, we explored incorporation of a positively charged PNA within DNA nanostructures. We investigated the efficiency of annealing a lysine-containing PNA probe with complementary, single-stranded DNA sequences within nanostructures, as well as the efficiency of duplex invasion and its dependence on salt concentration. Our results show that PNA allows for toehold-free strand displacement and that incorporation yield depends critically on binding site geometry. These results provide guidance for the design of PNA binding sites on nucleic acid nanostructures with an eye towards optimizing fabrication yield.

## 1. Introduction

During the last two decades, nucleic acids have emerged as a promising material for nanotechnology due to their highly predictable local geometry and programmable hybridization via Watson-Crick base-pairing. These well-understood molecular recognition rules and high-fidelity binding allow the formation of diverse structures through sequence design [1,2,3]. Of particular success has been an approach known as DNA origami in which a long single-stranded (scaffold) DNA is folded into a desired shape via a large number of short, synthetic (staple) strands [4,5,6]. Current methods for producing synthetic nucleic acids containing natural and/or artificial monomers with specific chemical modifications have enabled the preparation of milligram quantities of material. Such production levels are adequate for fundamental studies and practical applications [1]. The easy control of geometry, diverse range of shapes, and high resolution of the nucleic acid structures has led to an expansion of the field into the broader domain of biomaterials [7], finding use in tissue culture [8,9], drug delivery [10,11], immuno-technology [12,13,14] and intracellular imaging [15].

A crucial step in utilizing these structures is to expand their chemical versatility by developing covalent and non-covalent attachment strategies. In this way, DNA’s ability to self-assemble can be exploited for the nano-scale organization of diverse materials. For example, gold nanoparticles (AuNP) have been incorporated into structures through thiol groups introduced during DNA oligo synthesis, resulting in the capability to engineer both electronic and photonic properties [16,17,18,19,20,21].

DNA oligos modified with terminal amines or click-chemistry partners allow for chemical modification of resulting nanostructures with NHS-esters and through click chemistry [22]. Enzymatic modifications of DNA oligonucleotides with terminal transferase enable addition of deoxyribo-nucleotides to the 3′ ends of oligonucleotides [23]. In addition, the appended monomers can carry a wide variety of chemical groups that are thus displayed on the final assembled nanostructure [23]. In addition to chemical modification of DNA termini, internal purines have been sequence-specifically alkylated using pyrrole-imidazole polyamide enabling modification with functional groups such as biotin [24].

Proteins naturally display a range of chemical functionalities and catalytic activities and therefore are extremely desirable components to incorporate within or upon DNA nanostructures. Previously, it was shown that incorporation of antigens allows for the programmed patterning of antibodies on DNA nanostructures [25,26]. Another approach utilized DNA aptamers to attach proteins to DNA nanostructures [27]. The thiol modification used to link AuNPs to DNA has also been used for covalent coupling to amine groups using maleimide-*N*-hydroxysuccinimide hetero-bifunctional linkers [28]. The *N*-hydroxysuccinimide reactive group has been used to incorporate snap- and halo-tags allowing for covalent incorporation of fusion proteins into DNA nanostructures [29,30]. Further illustrating the diverse functionality of peptides, gold-binding peptides were covalently coupled to oligonucleotides and nanoscale patterning of AuNP was demonstrated without thiol chemistry [31].Sequence-specific binding of zinc-finger motif polypeptides have also been used to link fusion proteins to DNA-based nanostructures [32].

Unique among functionalization strategies is the use of peptide nucleic acids (PNA), which can bind to DNA in a construct via standard Watson-Crick base-pairing rules. PNA is a synthetic DNA analogue consisting of a backbone of repeating *N*-(2-aminoethyl)-glycine units to which purine and pyrimidine bases are linked via methylene carbonyl linkages [33]. The neutral backbone affords PNA physicochemical characteristics distinct from those of DNA, such as high affinity towards complementary, negatively-charged strands at low salt concentrations [34]. Since PNA is synthesized using the same automated, solid-phase chemistry as peptides, it is easy to make PNA-peptide chimeras, which in turn could be used to incorporate peptides at well-defined locations in DNA nanostructures [35].

**Figure 1:**
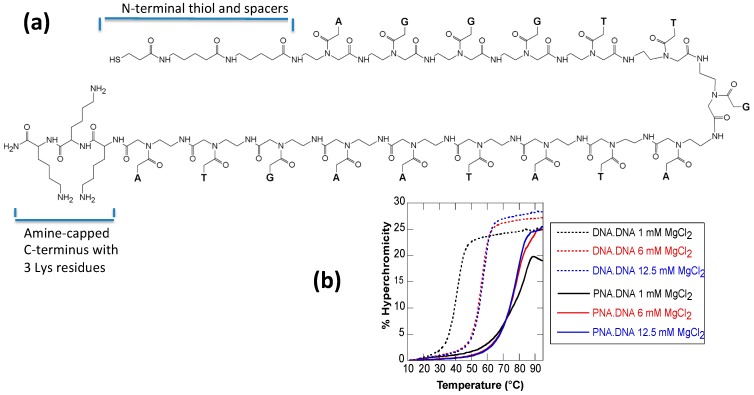
(**a**) Chemical structure of the PNA3K with nucleobases shown as single letters (A, T, G); (**b**) Melting curves of PNA3K·DNA heteroduplex (solid lines) and DNA·DNA homoduplex (dotted lines) at different concentrations of MgCl_2_.

The unique properties of PNA can also be used to construct nanostructures with different handedness than normal DNA duplexes [36] and with lower tolerance to base mismatches thus enabling high-fidelity detection systems [37,38]. One of the most interesting features of PNA molecules is that their high affinity for complementary DNA allows for sequence specific invasion into a DNA duplex. This property was used to create nanoscale assemblies using double-stranded DNA as a scaffold [39,40] and as a way to manipulate dynamic nanostructures by addition of PNA without predesigned toeholds [41,42]. Here we investigate the incorporation of PNA into DNA nanostructures under a variety of scenarios. We have designed a PNA strand (named PNA3K) composed of 16 nucleotides and three lysine residues at the C-terminal end (Figure 1a). The lysine sidegroups’ pK_a_ of approximately 10.5 adds three positive charges to the molecule. We investigate how PNA3K·DNA hybrids differ from DNA·DNA complex and especially how electrostatic charge might be used to maximize assembly yields for bottom-up nanofabrication.

## 2. Results and Discussion

### 2.1. Melting Curves

Since the relative stabilities of PNA·DNA heteroduplex and DNA·DNA homoduplex are known to change depending on ionic strength, we first assayed the melting temperature of PNA3K·DNA and DNA·DNA duplexes under different salt concentrations (Figure 1b). The melting curves were measured to evaluate thermodynamic stability of a DNA3K·PNA duplex compared to a DNA·DNA duplex. The DNA oligonucleotide sequence was either complementary or identical to that of the PNA. Enhanced thermal stability was observed for the heteroduplexes, with the melting temperatures (*T*_m_) of PNA3K·DNA duplex being above 75 °C for various salt concentrations. This is much higher (by 19–38 °C) than that of the DNA·DNA duplex (Table 1). This finding suggests that there can be a thermodynamic benefit of using PNA3K in DNA nanoassemblies. The melting temperatures of this 16-bp duplex are in general agreement with literature values of similar 15-bp duplexes without the three lysines [43].

**Table 1:** Melting temperatures (°C) of homo- and hetero-duplexes in 1× TAE buffer (40 mM Tris acetate, 2 mM EDTA at pH 8.0) and different concentration of MgCl_2_.

MgCl_2_ Concentration	DNA·DNA	PNA3K·DNA	Δ*T_m_*
1 mM	40	78	38
6 mM	56	77	21
12.5 mM	57	76	19

Representative melting curves are shown in Figure 1b. The melting curve of DNA·DNA duplex (dotted lines) has a slightly sharper transition around the melting temperature of duplex than the PNA3K·DNA heteroduplex (solid lines), which suggests a higher cooperativity of the base pairing in DNA·DNA duplex than in PNA3K·DNA hybrid. When the salt concentration is decreased from 12.5 mM to 1 mM, the thermal stability of PNA3K·DNA decreases only very slightly while that of DNA·DNA increases significantly. This observation demonstrates that there is a higher thermodynamic driving force for PNA3K binding to DNA at lower salt concentrations compared to the corresponding DNA·DNA binding. This observation is important since low salt conditions are frequently required for assembly steps involving metal nanoparticles. Since DNA nanostructures typically prefer high salt conditions, PNA-based adhesions may help alleviate these conflicting solution requirements.

### 2.2. Duplex Invasion

**Figure 2:**
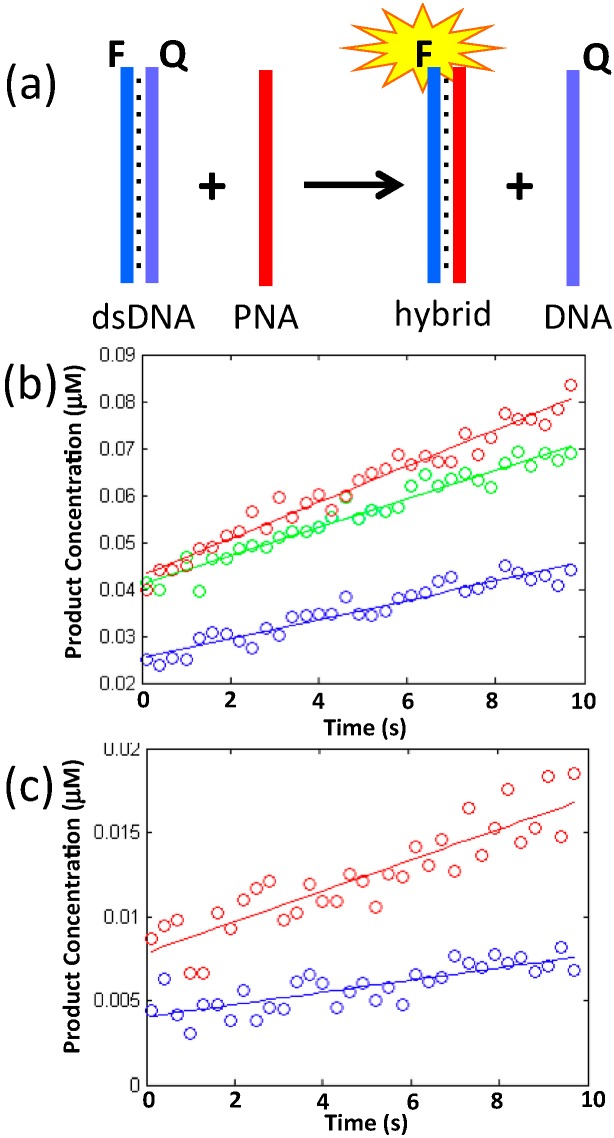
(**a**) Schematic representation of the fluorescence-based duplex invasion assay. PNA3K displaces the quencher-labeled DNA strand and turns on the fluorescent signal; (**b**) Graph of product concentration *vs.* time for PNA3K-mediated DNA duplex dissociation at the following starting concentrations: Blue, 0.5 μM duplex and 1 μM PNA3K; Green, 0.5 μM duplex and 2 μM PNA3K; Red, 1 μM duplex and 2 μM PNA3K; (**c**) DNA duplex dissociation at different MgCl_2_ concentrations: Red, 6 mM MgCl_2_ and Blue, 12.5 mM MgCl_2_.

The assay described above examined the binding of PNA to complementary single-strand DNA, however PNA has also been shown to be capable of “invading” double-strand DNA and displacing one DNA strand to form a stable heteroduplex with the other [39,40,41,42,43]. We have evaluated the kinetics of strand invasion of PNA3K into a DNA duplex along with its dependence on salt concentration. Before investigating the propensity of our positively-charged PNA3K probe to invade a duplex embedded in a complicated nanostructure, we first performed a fluorescence assay of the invasion into a simple DNA duplex labeled with a fluorophore and quencher pair (Figure 2). Using this data and the method of initial rates, we isolated the exponents in the rate equation; $v=k{\left[DNA\right]}^{X}{\left[PNA3K\right]}^{Y}$ by performing three different experiments with two different concentrations of DNA and PNA3K:

(1) $$v_{1}=k{{\left[DNA\right]}_{1}}^{X}{{\left[PNA3K\right]}_{1}}^{Y}$$

(2) $$v_{2}=k{{\left[DNA\right]}_{2}}^{X}{{\left[PNA3K\right]}_{2}}^{Y}$$

(3) $$v_{3}=k{{\left[DNA\right]}_{1}}^{X}{{\left[PNA3K\right]}_{2}}^{Y}$$

The exponents for both PNA3K and DNA could then be isolated in the following way;

(4) $$\frac{\nu_{1}}{\nu_{3}}=\frac{k{{\left[DNA\right]}_{1}}^{X}{{\left[PNA3K\right]}_{1}}^{Y}}{k{{\left[DNA\right]}_{1}}^{X}{{\left[PNA3K\right]}_{2}}^{Y}}$$

(5) $$Y=\frac{\ln\frac{\nu_{1}}{\nu_{3}}}{\ln\frac{{\left[PNA3K\right]}_{1}}{{\left[PNA3K\right]}_{2}}}$$

Here, [DNA] is the concentration of pre-annealed DNA duplex and [PNA3K] is the concentration of added PNA3K. Figure 2 shows representative graphs of the initial concentration of dissociated duplex following addition and mixing of PNA3K in solution containing 1 mM MgCl_2_. Isolating the exponents results in values of 0.34 and 0.72 for X and Y, respectively. Rounding off to the nearest fractions, we get the following rate equation $r=k{\left[DNA\right]}^{1/3}{\left[PNA3K\right]}^{2/3}$, which corresponds to a ratio of 2:1 PNA3K to DNA in the rate limiting step in accordance with previous reports on the invasion of poly-T PNA oligos into much longer DNA duplexes [43]. Additional experiments will be required before we can fully understand the detailed mechanism of PNA3K strand invasion.

We also examined the affect of varying MgCl_2_ concentration on PNA3K-induced DNA dissociation. Figure 2c shows representative graphs of the initial rates of duplex dissociation. Using the exponents determined above, we calculated the rate constants at different salt concentrations (Table 2).

**Table 2:** Dissociation rate constants at different MgCl_2_ concentrations.

	1 mM MgCl_2_	6 mM MgCl_2_	12.5 mM MgCl_2_
k (s^−1^∙M^−1^)	0.0025	0.00072	0.00034

We observed a decrease in the rate constant of almost an order of magnitude when going from 1 mM MgCl_2_ to 12.5 mM MgCl_2_; the latter is the concentration of Mg^2+^ usually used for annealing DNA nanostructures. The decrease in invasion rate with increased ionic strength is the result of increased stability of the DNA duplex and correspondingly slower PNA3K-induced DNA dissociation.

**Figure 3:**
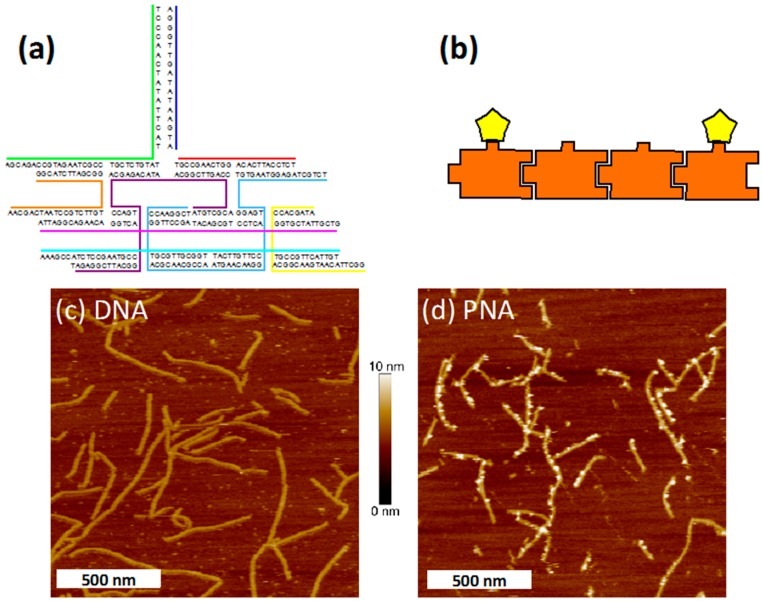
(**a**) Structure and sequence of the TX tile. The top protruding duplex provides a site for strand invasion of complementary PNA; (**b**) Schematic drawing of four TX tiles assembled into a linear array by sticky-end cohesion. Visualization of PNA3K invasion was achieved by incubating the arrays with biotinylated PNA3K (bPNA3K) and then with streptavidin (SA) since bound SA can be imaged by AFM. In this arbitrary example, SA protein (yellow pentagons) is shown bound to the first and last tiles in a four-tile array with the middle two binding sites shown unoccupied; (**c**) AFM image of TX arrays incubated with biotinylated DNA in 1 mM MgCl_2_. No SA is visible on the arrays, indicating that no binding of the biotinylated DNA occurred; (**d**) AFM image of TX arrays incubated in buffer containing 1 mM MgCl_2_, bPNA3K, and SA. Bound SA is observed, indicating successful strand invasion by PNA3K into the DNA duplexes on the DNA tiles.

Utilizing this information, we annealed linear TX tile arrays [44,45] decorated with duplex invasion sites (Figure 3a,b) to which PNA could bind. Biotinylated PNA was used so that its binding could be observed via atomic force microscopy (AFM) imaging after streptavidin (SA) protein was allowed to bind to the biotin moieties (Figure 3d). To begin, the TX arrays were prepared by annealing in the presence of 12.5 mM MgCl_2_ and then buffer exchanged into 1 mM MgCl_2_ (see Experimental Section). Next, biotinylated PNA3K (bPNA3K) was added so that the PNA concentration was equimolar with the invasion sites within the TX arrays. This solution was incubated for >2 h before deposition on mica under buffer containing 12.5 mM MgCl_2_ and 1–2 μM SA and finally imaged by AFM. The control sample with biotinylated DNA added, showed no SA localization to the arrays (no invasion) (Figure 3c). In contrast, the sample incubated with biotinylated PNA showed the protein localized on the DNA array similar to beads on a string (Figure 3d).

### 2.3. Binding of PNA to DNA Origami

**Figure 4:**
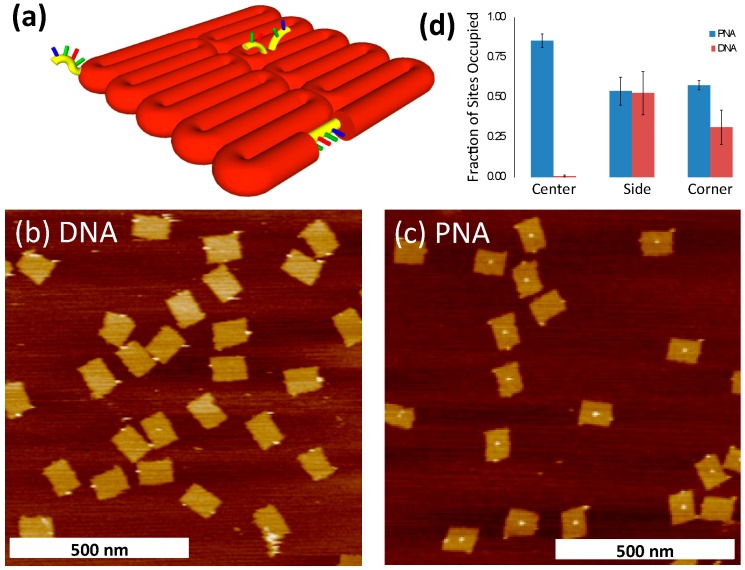
(**a**) Schematic drawing of modified origami displaying three different PNA3K binding sites. Orange cylinders represent DNA duplex folded into the origami. Yellow cylinders represent single-stranded DNA regions complementary to PNA3K at the three binding sites (corner, center, and side). Representative AFM images of the origami with biotinylated DNA (**b**) and biotinylated PNA (**c**); (**d**) Fraction of sites to which SA was bound for PNA (blue) or DNA (red). Each micrograph was examined and tallied individually, and the mean proportion of SA occupied sites (binding events) is displayed with error bars representing standard deviation.

Next, we examined incorporation of bPNA3K compared to DNA during the annealing of DNA origami at a variety of different binding sites. We modified a rectangular origami structure of Rothemund’s original design [4] by adding three binding sites for the bPNA3K at different locations and configurations (Figure 4a). We refer to these three sites by their locations as “side”, “center”, and “corner”. The side site consists of a stretch of natural M13 sequence that is complementary to the bPNA3K sequence and is exposed as single-strand scaffold DNA along the side of the rectangular structure. The center site is situated at the geometric center of the two-dimensional origami sheet and consists of overhangs from two separate staple strands each complementary to half of the bPNA3K molecule, thus forming a V-shaped binding site for the PNA. The central V-site design follows from similar RNA binding sites described previously [46]. The corner site is located at one of the four corners and consists of a single-stranded overhang extending from a staple strand and with a sequence fully complementary to the bPNA3K molecule. Incorporation of bPNA3K at the various sites was assayed by binding streptavidin (SA) and imaging by AFM (Figure 4).

Origami samples were annealed with a concentration of bPNA3K equivalent to that of the binding sites, and then incubated with 1–2 μM SA for 1–2 h and imaged by AFM. Figures 4a,b show representative images of structures with biotinylated DNA (bDNA) or bPNA3K, respectively. A total of 10 micrographs each were collected and analyzed for SA binding (indicative of bDNA or bPNA3K binding) at each of the three sites (see Figures S1–S20). The number of structures and binding events observed are listed in Table 3. The fraction of sites to which bound SA was observed was calculated for each individual micrograph. The mean fraction bound for each type of binding site for PNA and DNA are displayed in Table 3 and Figure 4d.

**Table 3:** Total number of counted origami structures and binding events.

	bPNA3K	DNA
Origami	596	883
Center site	510	5
Side site	322	458
Corner site	344	276

The AFM image in Figure 4c clearly shows that the PNA effectively binds to the center site of the origami; indeed a significantly higher fraction of occupied binding sites is observed compared to the two other sites. On the other hand, the 16 nucleotide bDNA strand, showed almost zero binding at the center site. This is in agreement with previous reports of diminished hybridization at the center of the origami which Jungmann *et al.* ascribed to an increased off rate [47]. We ascribe the very low level of binding observed here to the combination of the location on the origami (surrounded by negative charges on the DNA backbone) and the steric properties of the center binding site. The V-shaped binding conformation was originally introduced by Ke *et al*. to aid in the assessment of binding of 40 nt long probes via AFM, but this conformation appears to be ill suited for shorter DNA probes [46]. In addition to decreased binding (on rate) due to DNA/DNA electrostatic repulsion, it is conceivable that fraying of duplex originating at the V vertex can quickly expand to full dissociation (increased off rate). Interestingly, we do see a higher binding fraction of the DNA control probe to the side site compared to the corner site. We ascribe this to the additional base stacking energy gained at the ends of the DNA probe, where the probe tightly abuts neighbouring nucleobase pairs. Including base stacking interactions at the ends of probe target sites might be a good strategy for increasing binding affinities of future binding site designs. The positively charged bPNA3K strand shows a higher frequency of binding to the corner site than the DNA probe, but the PNA and DNA showed about the same degree of binding to the side site. This could be due to steric issues at the ends of the bPNA3K resulting from the lysine residues at the C-terminal end and the C10 spacer on the *N*-terminus that could affect the nearby base-stacking interactions. The biggest difference between PNA and DNA binding is observed at the center site (almost no bDNA binding but bPNA3K binding to about 85% of the sites). This could be due to the difference in the electrostatic interactions between the bPNA3K probe and the origami surface and the corresponding interactions between the bDNA probe and the origami surface. Unlike the corner site that protrudes freely into solution and can thus avoid significant contact with other parts of the structure and the side site that “sees” only one neighboring duplex, the center site sits in the middle of the negatively charged origami. The conformation of the center binding site is such that the entirety of the probe is brought very close to the origami surface, thus increasing the importance of electrostatic attraction and repulsion. Johnson-Buck *et al*. proposed that DNA nanostructures enrich the immediate surrounding environment in positive counterions at low ionic strength [48]. Our present results suggest that such enrichment is insufficient to prevent fraying of the DNA probe hybridization at the binding site vertex, leading to dissociation. On the other hand, the positively charged bPNA3K is highly attracted to the origami surface and binds efficiently.

The charge of the DNA backbone represents a challenge in the self-assembly of large, densely-packed, three-dimensional nanostructures which can require slow anneals of up to 178 h with high salt concentrations [49,50]. Our results suggest that positively-charged PNA probes of even shorter lengths could be tested as “anchor” strands enabling the modification of relatively large DNA structures relatively quickly in shorter, more convenient annealing steps.

## 3. Experimental Section

### 3.1. PNA Synthesis

PNA oligomers were synthesized using standard solid-phase peptide synthesis methods with a Boc-protection strategy [51]. MBHA resin was preloaded with Boc-l-Lys-(4-MeOBzl)-OH (NovaBiochem) to an estimated loading of 0.05 mequiv/g. Boc group on resin is deprotected with 5% *m*-cresol in trifluoroacetic acid (TFA). According to the sequence of the PNA oligomer, a Boc-protected PNA monomer (A, G, T, or C) (ASM Research Chemicals) was coupled to the resin using *O*-Benzotriazole-*N*,*N*,*N*′,*N*′-tetramethyluronium hexafluoro-phosphate (HBTU) (Peptides International) as a coupling agent. Unreacted -NH_2_ sites were capped by acetic anhydride. Finally, 5-aminovaleric acid (C_4_) as a linker and 3-mercaptopropionic acid (C_2_) as a source of -SH group were coupled at the end of the PNA oligomer to allow for functionalization with biotin. PNA oligomers were cleaved from the resin with TFA and trifluoromethanesulfonic acid (TFMSA), precipitated in diethyl ether, and dried with a stream of nitrogen. PNA oligomers were purified by reverse-phase chromatography using a C18 silica column on a Waters 600 HPLC. Absorbance was measured at 260 nm with a Waters 2996 Photodiode Array Detector. Characterization of the oligomers was performed by MALDI-ToF mass spectrometry on an Applied Biosystems Voyager Biospectrometry Workstation with Delayed Extraction and an R-cyano-4-hydroxycinnamic acid matrix (10 mg/mL in 1:1 water/acetonitrile, 0.1% TFA). The sequence of the PNA synthesized for this study was Lys_3_-ATGAATATAGTTGGGA-C_4_C_4_C_2_. PNA solutions were prepared in nanopure water (>18.3 MΩ∙cm^−1^) and stored at −18 °C to avoid depurination reactions. The concentration of PNA stock solution was determined by UV-Vis spectrophotometry using ε(260) = 6600, 8600, 11,700, and 13,700 cm^−1^∙M^−1^ for each C, T, G, and A monomer, respectively. For biotinylation the PNA was first reduced using immobilized TCEP (Thermo Scientific, Waltham, MA, USA) and then biotinylated with EZ-Link Maleimide-PEG2-Biotin2 (Thermo Scientific). The final product was purified by reverse phase HPLC.

### 3.2. UV-Vis Melting Curves

UV melting curves were recorded at 260 nm in the temperature range of 10–95 °C for both cooling and heating modes, at the rate of 1 °C/min. Prior to the measurement of the melting profiles, solutions of 3 μM of each ssPNA in 1× TAE and different concentrations of MgCl_2_ were kept at 95 °C for 10 min before being slowly cooled. *T*_m_ is the inflection point of a sigmoidal function used to fit the melting curve.

### 3.3. Nanostructure Assembly

All DNA strands were purchased from Integrated DNA Technologies (IDT) (Coralville, IA, USA). TX arrays were annealed at 200 nM of each strand by slowly cooling from 90 to 20 °C over 8 h. Following annealing, the sample was buffer exchanged into TAE buffer (Tris, 40 mM; acetic acid, 20 mM; EDTA, 2 mM; pH 8.0) containing 1 mM MgCl_2_ at 4 °C. PNA or DNA control oligo was then added to 200 nM and incubated for at least 2 h, then brought to 12.5 mM MgCl_2_ before imaging with AFM. Origami samples were annealed from 90 to 20 °C over 2 h in TAE/Mg^2+^ (Tris, 40 mM; acetic acid, 20 mM; EDTA, 2 mM; and magnesium acetate, 12.5 mM; pH 8.0) with 5 nM M13 scaffold, 50 nM of each staple strand, and 200 nM PNA or DNA control strands. Following annealing, the excess staple strands were removed using Microcon 100,000 MWCO spin filter units at 4 °C and subsequently kept on ice until prepped for AFM.

### 3.4. Atomic Force Microscopy

Origami samples were mixed with 1–2 μM streptavidin (SA) protein and incubated at room temperature for 1–2 h. Then, a 5 μL sample was deposited on freshly cleaved mica. TX array samples were put directly on freshly cleaved mica and then SA was added to a concentration of 1–2 μM. AFM images were obtained on a Digital Instruments Nanoscope III (Vecco, Plainview, NY, USA) using NP-S oxide-sharpened silicon nitride tips (Vecco) with a multimode head and fluid cell in tapping mode under TAE/Mg buffer.

### 3.5. Fluorescence

Fluorescence data were collected using a Cary Eclipse Fluorescence Spectrophotometer from Varian, with temperature controller set to 23 °C. Excitation and emission wavelengths were 648 and 668 nm with a 5 nm slitwidth. Complementary DNA strands with fluorophore “Cy5” and quencher “Iowa Black RQ” were purchased from IDT and pre-annealed in TAE buffer with specified MgCl_2_ concentration by incubating at 90 °C for 5 min and then cooling to room temperature. PNA3K was added and mixed within a 10 s period and data points were recorded every 0.1 s and subsequently groups of 3 data points were averaged. Fluorescence data was transformed to concentration values by setting the base level to 0 μM dissociated duplex and recording maximum fluorescence following thermal melting and annealing of duplex with PNA3K and equating this to the original concentration of duplex. A 10 s period following mixing was used to fit a linear equation giving the initial rate of reaction. All rates were measured in triplicate and the average used to calculate the exponents in the rate equation (see above).

## 4. Conclusions

One previous study has been reported in which PNA strands were bound to DNA origami structures, however, in that case, pyrimidine-rich PNA was used to form triplex with DNA in order to break up specific DNA·DNA interactions [42]. Here, we examined and compared a variety of binding site geometries with an eye toward using PNA·DNA interactions to facilitate nanofabrication. We have shown that PNA molecules bearing positively charged Lys residues behave differently than DNA when interacting with DNA nanostructures as evidenced by the altered binding distribution of the PNA *vs.* DNA probes on a two-dimensional DNA origami. Compared to negatively charged DNA probes, the PNA probes bound more effectively to the center of DNA origami structures where there is a high density of negative charge. This feature can be exploited in the design and fabrication of site-specifically modified DNA-based materials for nanotechnology and biotechnology applications. Furthermore, the use of PNA increases the toolbox for creating dynamic nanoscale molecular devices. Additional research is needed to better understand and model the mechanism of binding of modified PNA oligos to DNA origami structures in order to create accurate assembly and predictable function in artificial nanoscale machinery.

## Supplementary Material

Supplementary materials can be accessed at: http://www.mdpi.com/1420-3049/20/09/17645/s1.
