# Supplementary material for: Comparative Incorporation of PNA into DNA Nanostructures
Source: Molecules. 2015 Sep 23;20(9):17645–58. doi: 10.3390/molecules200917645 (PMC6331967; doi:10.3390/molecules200917645)
Supplement: Supplementary file 1 [file molecules-20-17645-s001.pdf]

Supplementary Materials

1. AFM Micrographs

Fully formed structures wholly within each micrograph were counted. Structures not fully formed were not counted and are marked with a yellow cross. Center located streptavidin is marked with a red circle, side located with a blue circle and corner located with a green circle.

bPNA3K Incorporated in Origami (Figures S1–S10).

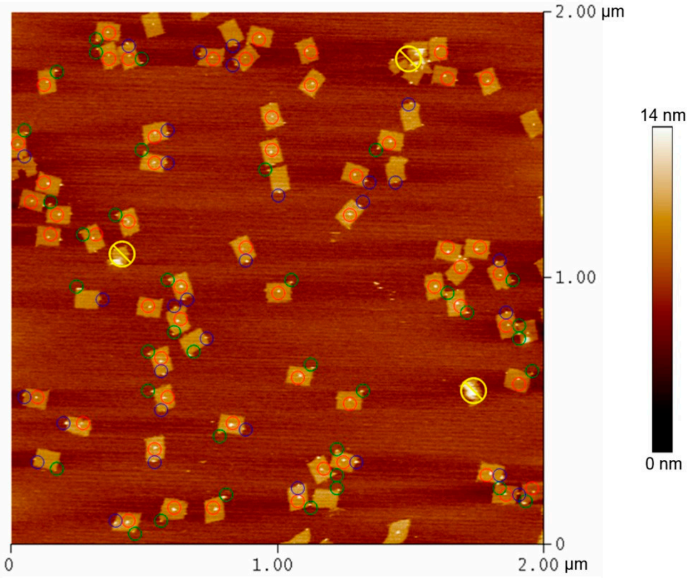

Figure S1. AFM image #1 of DNA origami with incorporated bPNA3K. Structures not fully formed were not counted and are marked with a yellow slash. Streptavidin located at center sites are marked with a red circle, located at side sites with a blue circle, and corner sites with a green circle.

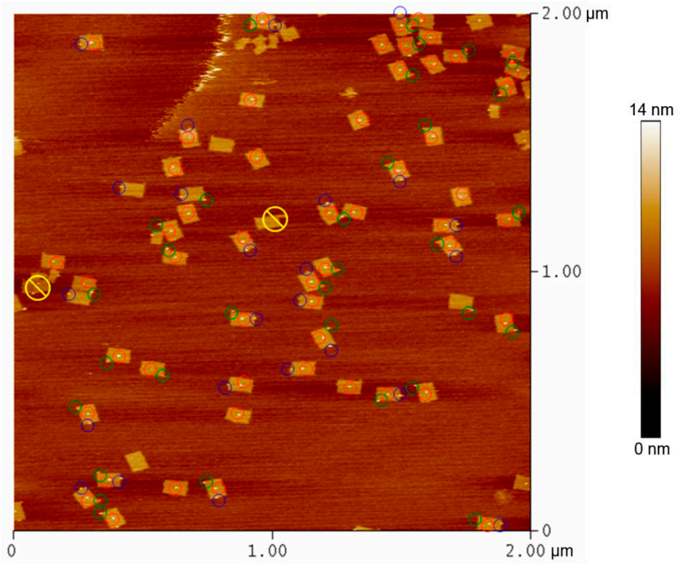

Figure S2. AFM image #2 of DNA origami with incorporated bPNA3K. Structures not fully formed were not counted and are marked with a yellow slash. Streptavidin located at center sites are marked with a red circle, located at side sites with a blue circle, and corner sites with a green circle.

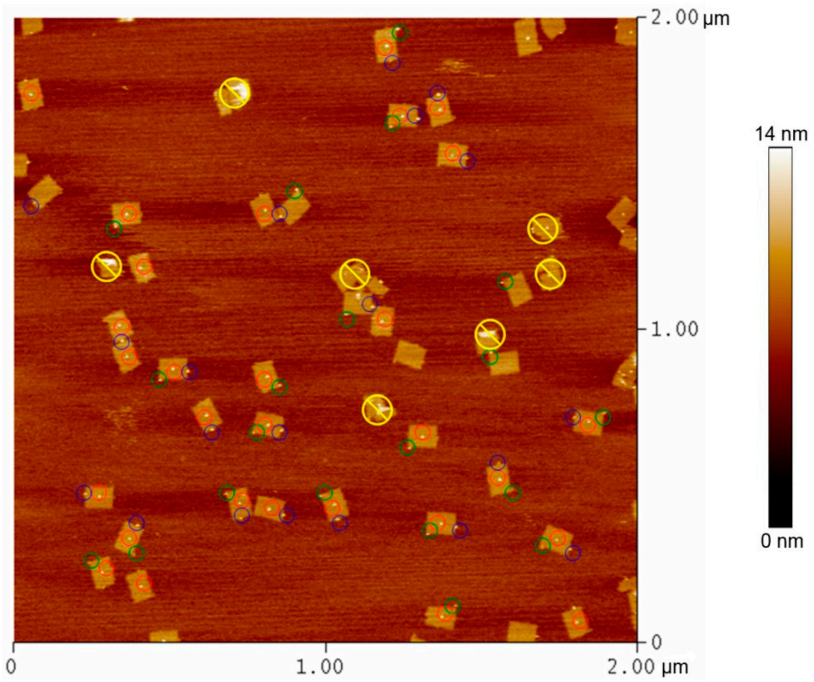

Figure S3. AFM image #3 of DNA origami with incorporated bPNA3K. Structures not fully formed were not counted and are marked with a yellow slash. Streptavidin located at center sites are marked with a red circle, located at side sites with a blue circle, and corner sites with a green circle.

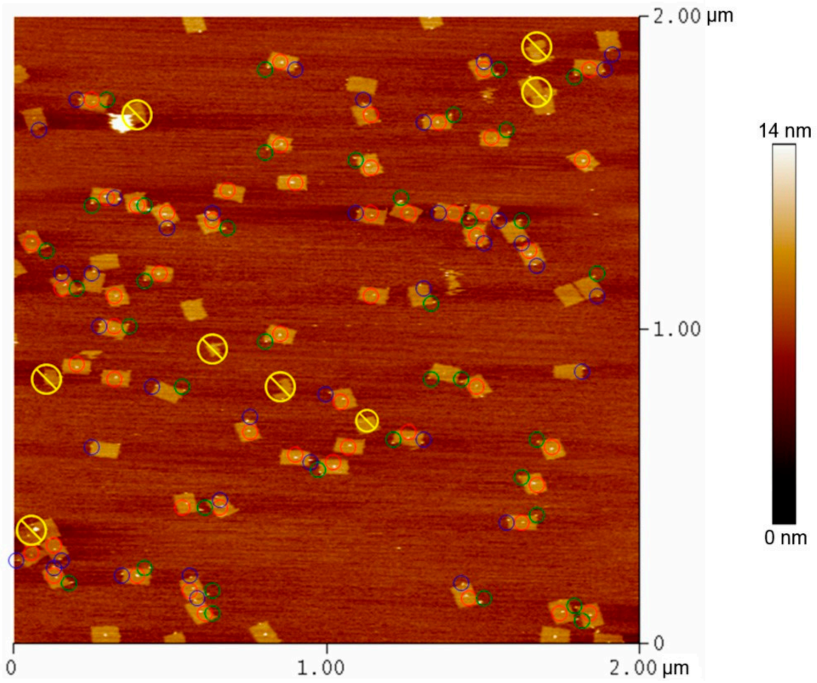

Figure S4. AFM image #4 of DNA origami with incorporated bPNA3K. Structures not fully formed were not counted and are marked with a yellow slash. Streptavidin located at center sites are marked with a red circle, located at side sites with a blue circle, and corner sites with a green circle.

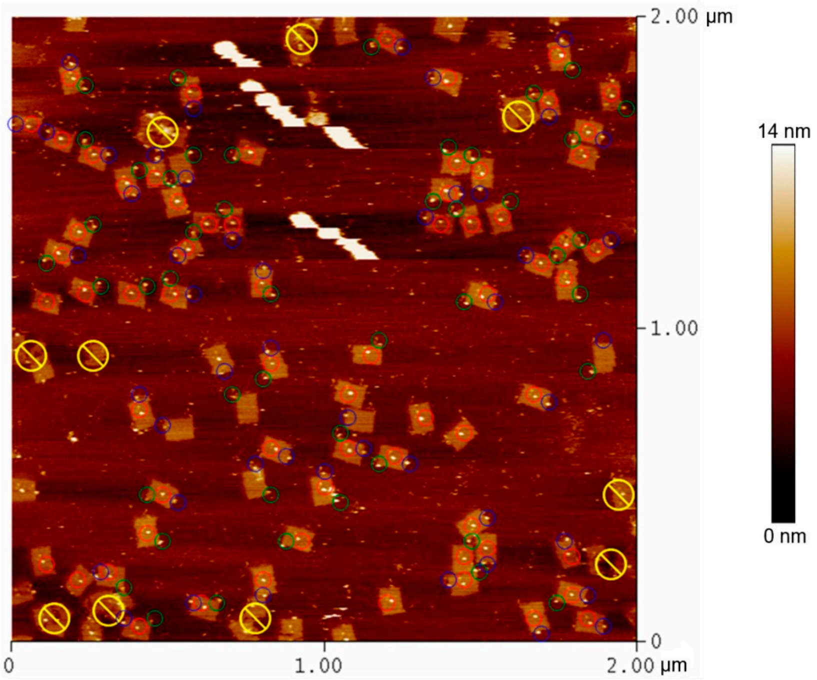

Figure S5. AFM image #5 of DNA origami with incorporated bPNA3K. Structures not fully formed were not counted and are marked with a yellow slash. Streptavidin located at center sites are marked with a red circle, located at side sites with a blue circle, and corner sites with a green circle.

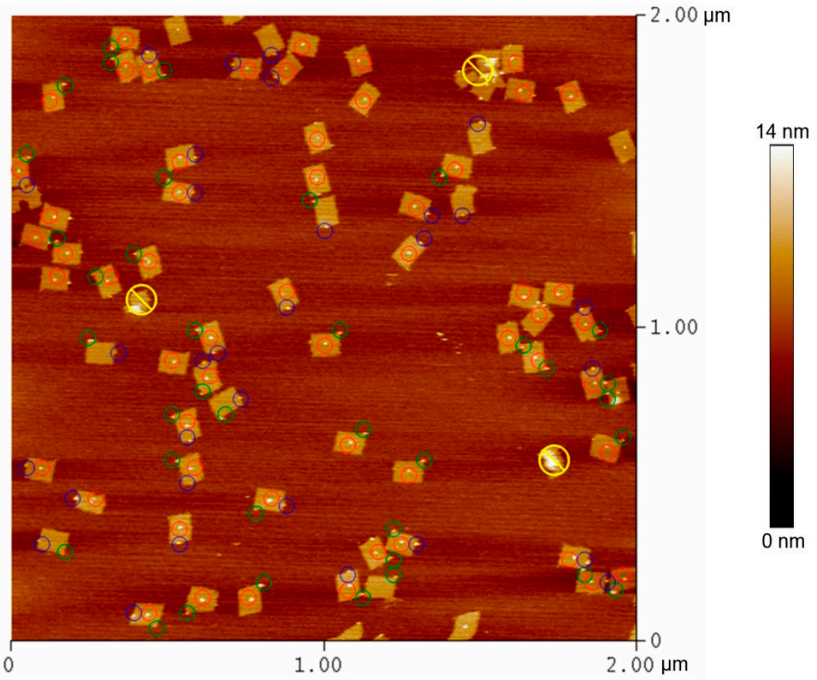

Figure S6. AFM image #6 of DNA origami with incorporated bPNA3K. Structures not fully formed were not counted and are marked with a yellow slash. Streptavidin located at center sites are marked with a red circle, located at side sites with a blue circle, and corner sites with a green circle.

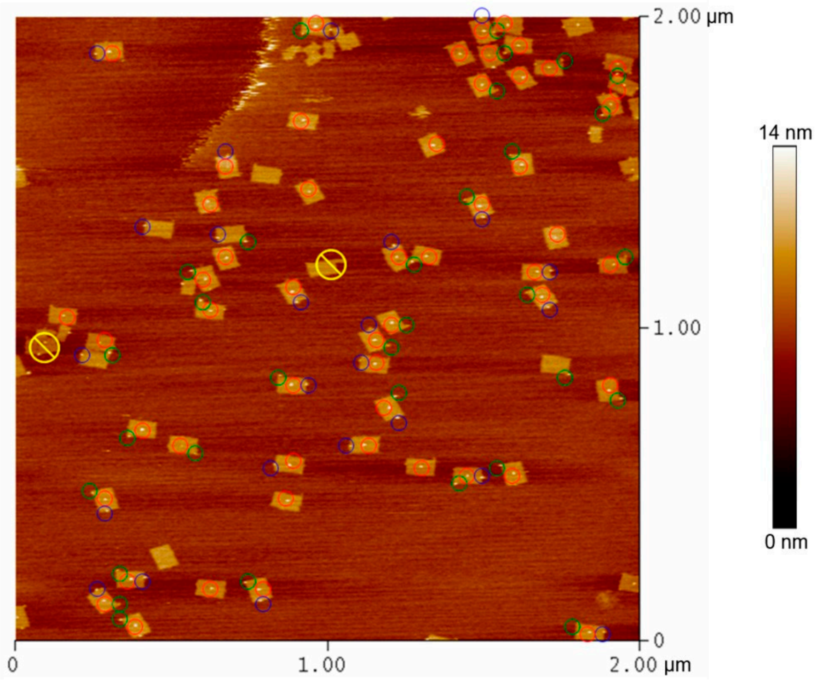

Figure S7. AFM image #7 of DNA origami with incorporated bPNA3K. Structures not fully formed were not counted and are marked with a yellow slash. Streptavidin located at center sites are marked with a red circle, located at side sites with a blue circle, and corner sites with a green circle.

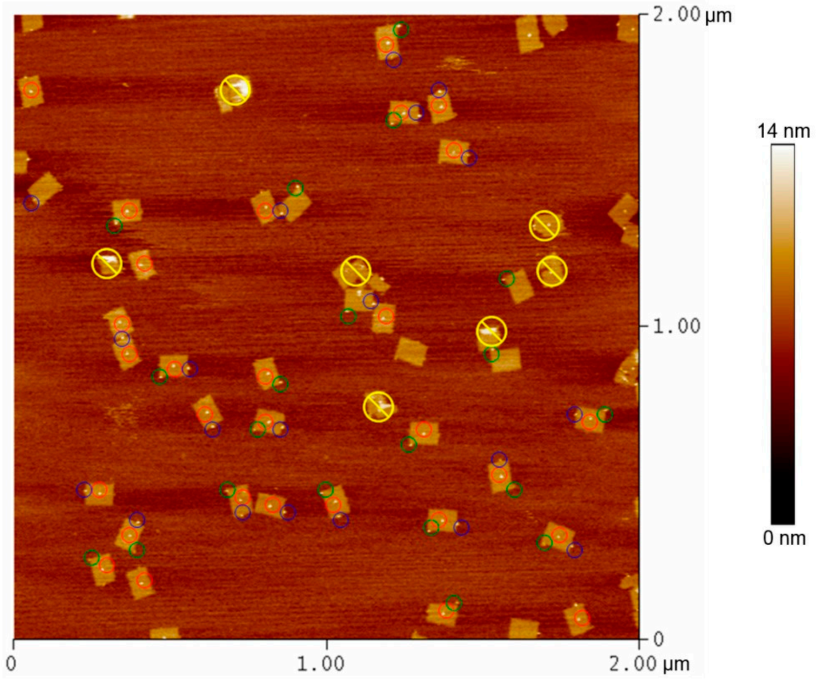

Figure S8. AFM image #8 of DNA origami with incorporated bPNA3K. Structures not fully formed were not counted and are marked with a yellow slash. Streptavidin located at center sites are marked with a red circle, located at side sites with a blue circle, and corner sites with a green circle.

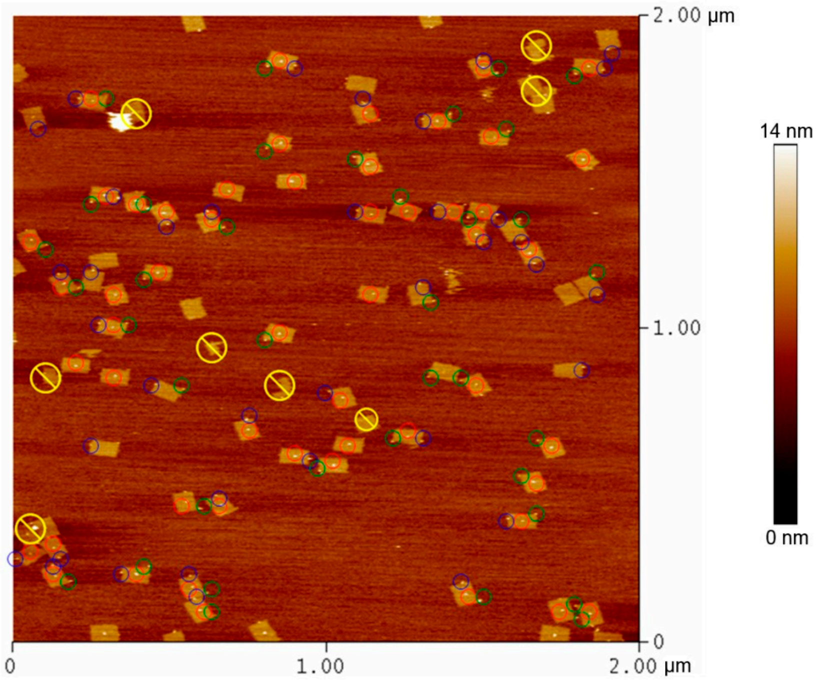

Figure S9. AFM image #9 of DNA origami with incorporated bPNA3K. Structures not fully formed were not counted and are marked with a yellow slash. Streptavidin located at center sites are marked with a red circle, located at side sites with a blue circle, and corner sites with a green circle.

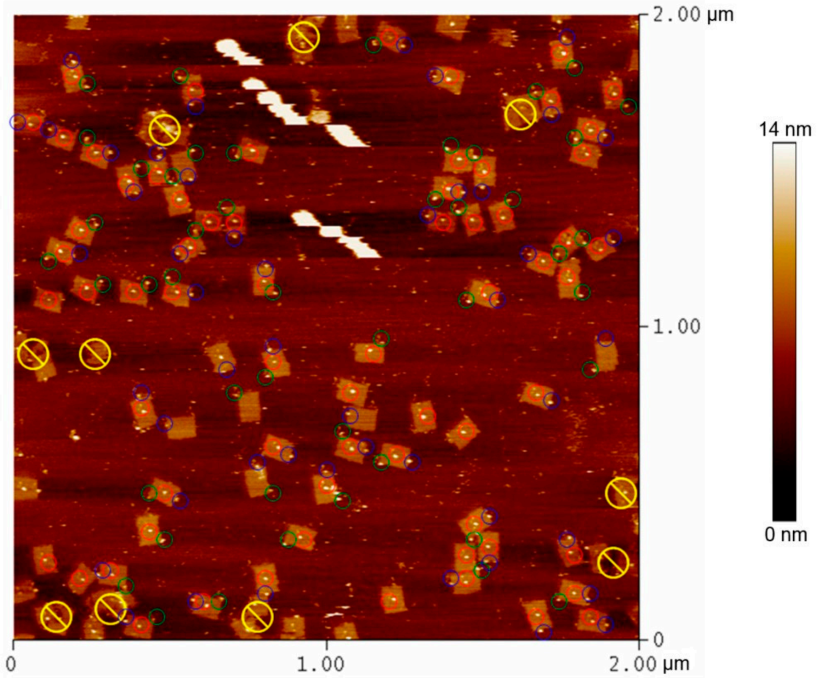

Figure S10. AFM image #10 of DNA origami with incorporated bPNA3K. Structures not fully formed were not counted and are marked with a yellow slash. Streptavidin located at center sites are marked with a red circle, located at side sites with a blue circle, and corner sites with a green circle.

Biotinylated DNA (bDNA) Control Incorporated in Origami (Figures S11–S20)

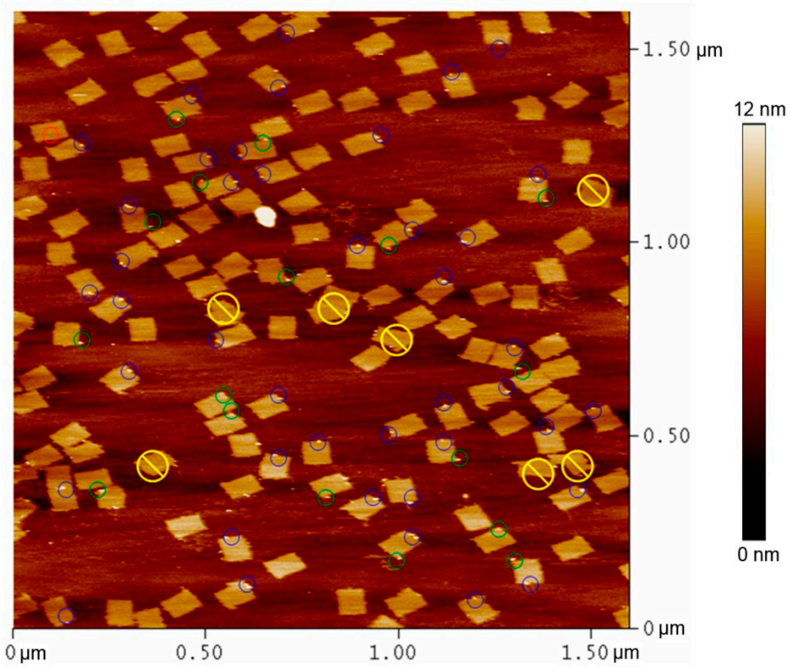

Figure S11. AFM image #1 of DNA origami with incorporated bDNA. Structures not fully formed were not counted and are marked with a yellow slash. Streptavidin located at center sites are marked with a red circle, located at side sites with a blue circle, and corner sites with a green circle.

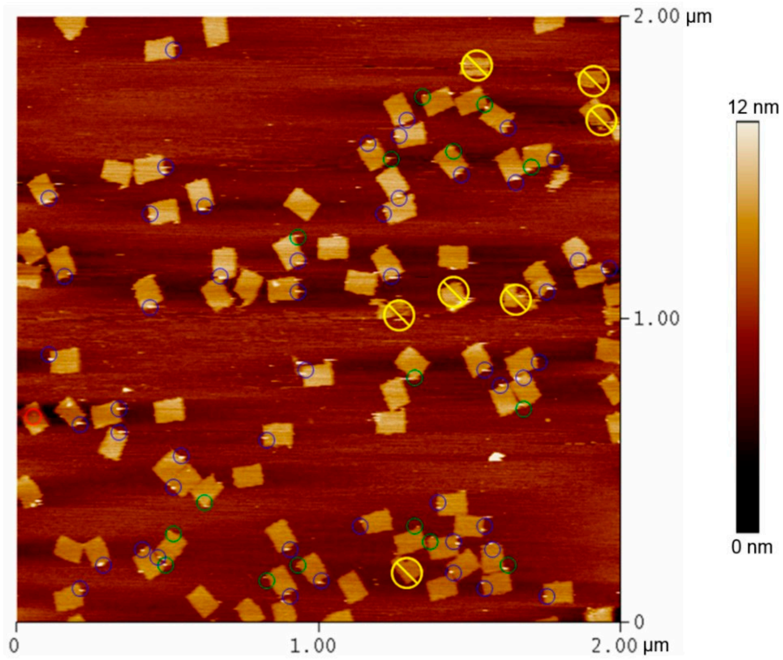

Figure S12. AFM image #2 of DNA origami with incorporated bDNA. Structures not fully formed were not counted and are marked with a yellow slash. Streptavidin located at center sites are marked with a red circle, located at side sites with a blue circle, and corner sites with a green circle.

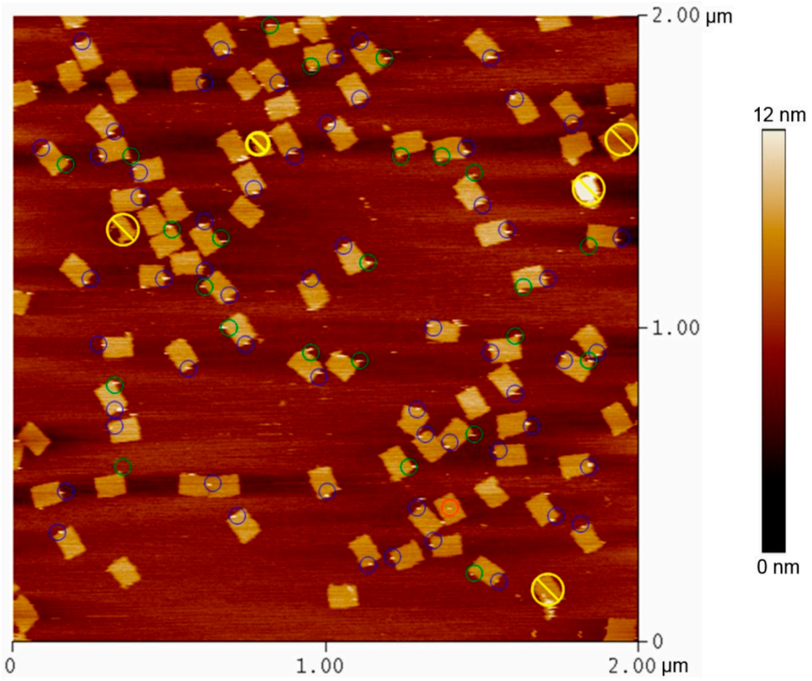

Figure S13. AFM image #3 of DNA origami with incorporated bDNA. Structures not fully formed were not counted and are marked with a yellow slash. Streptavidin located at center sites are marked with a red circle, located at side sites with a blue circle, and corner sites with a green circle.

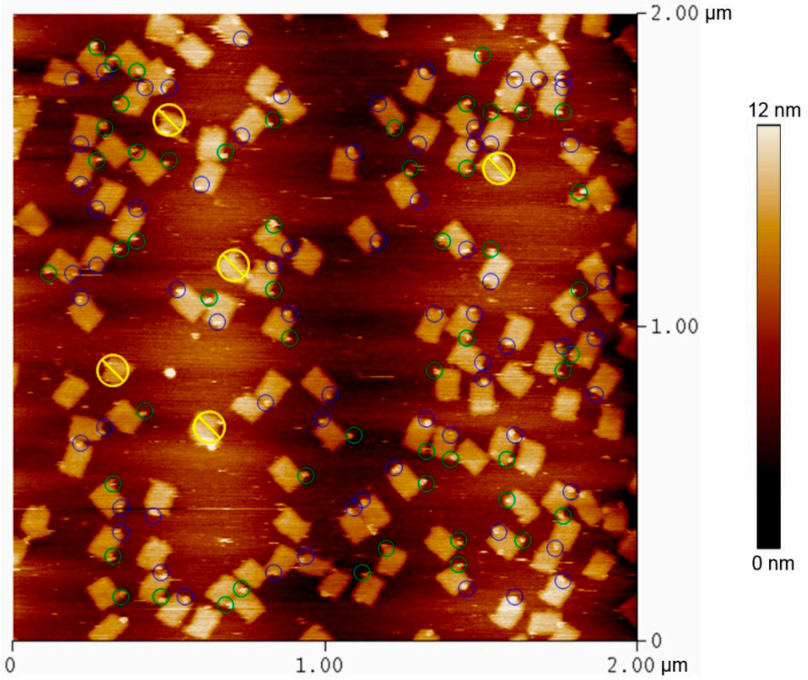

Figure S14. AFM image #4 of DNA origami with incorporated bDNA. Structures not fully formed were not counted and are marked with a yellow slash. Streptavidin located at center sites are marked with a red circle, located at side sites with a blue circle, and corner sites with a green circle.

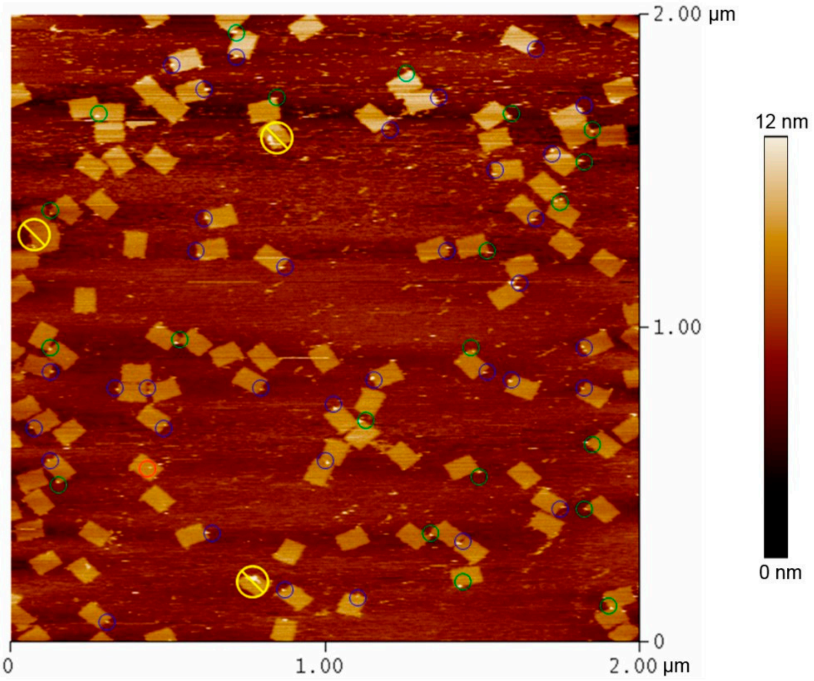

Figure S15. AFM image #5 of DNA origami with incorporated bDNA. Structures not fully formed were not counted and are marked with a yellow slash. Streptavidin located at center sites are marked with a red circle, located at side sites with a blue circle, and corner sites with a green circle.

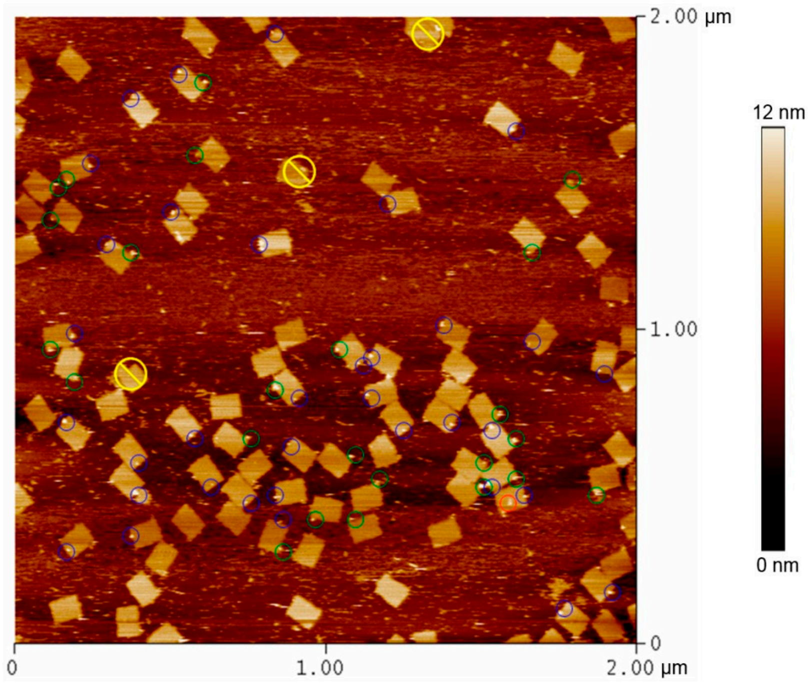

Figure S16. AFM image #6 of DNA origami with incorporated bDNA. Structures not fully formed were not counted and are marked with a yellow slash. Streptavidin located at center sites are marked with a red circle, located at side sites with a blue circle, and corner sites with a green circle.

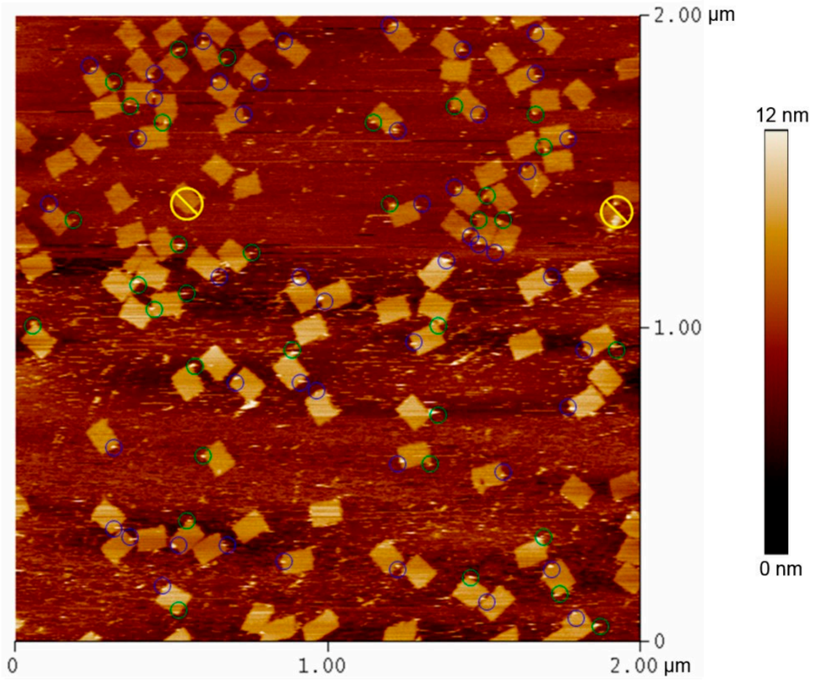

Figure S17. AFM image #7 of DNA origami with incorporated bDNA. Structures not fully formed were not counted and are marked with a yellow slash. Streptavidin located at center sites are marked with a red circle, located at side sites with a blue circle, and corner sites with a green circle.

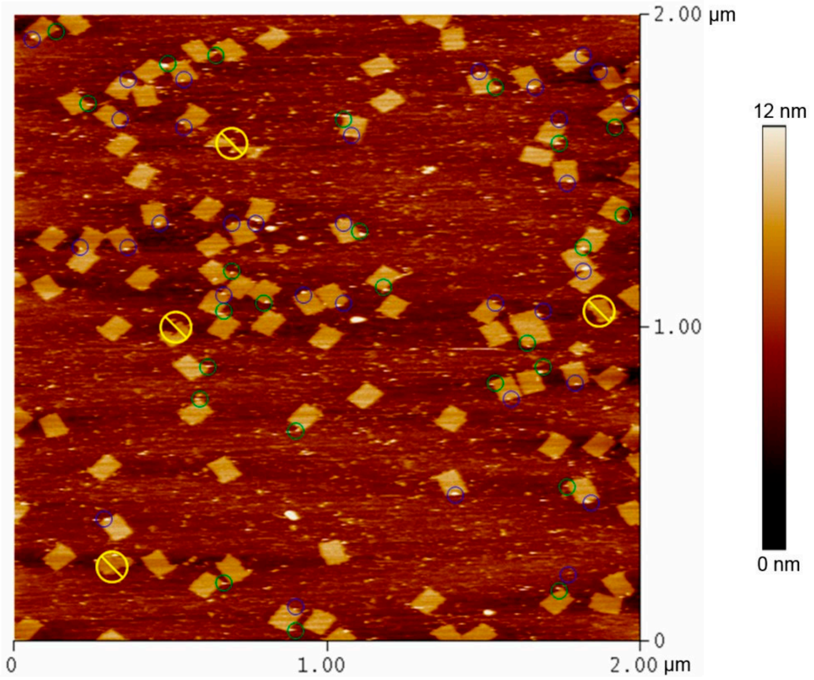

Figure S18. AFM image #8 of DNA origami with incorporated bDNA. Structures not fully formed were not counted and are marked with a yellow slash. Streptavidin located at center sites are marked with a red circle, located at side sites with a blue circle, and corner sites with a green circle.

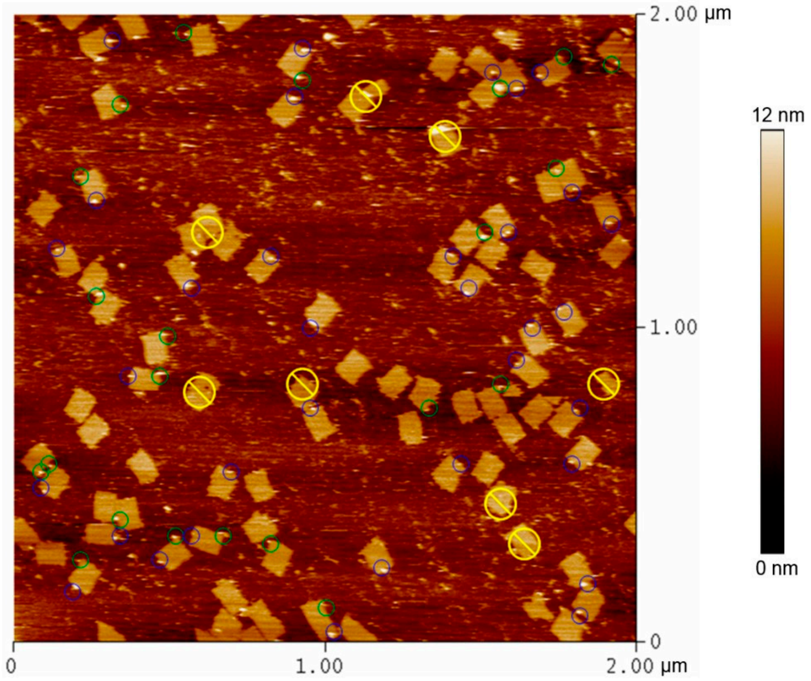

Figure S19. AFM image #9 of DNA origami with incorporated bDNA. Structures not fully formed were not counted and are marked with a yellow slash. Streptavidin located at center sites are marked with a red circle, located at side sites with a blue circle, and corner sites with a green circle.

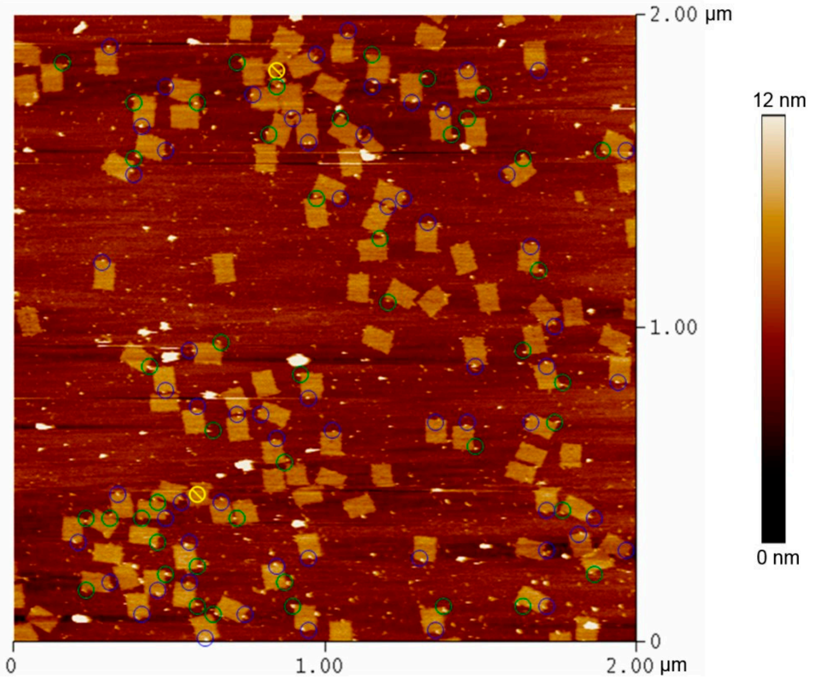

Figure S20. AFM image #10 of DNA origami with incorporated bDNA. Structures not fully formed were not counted and are marked with a yellow slash. Streptavidin located at center sites are marked with a red circle, located at side sites with a blue circle, and corner sites with a green circle.

2. DNA Sequences (5′–3′)

2.1. Center Strands

t-1r16e and t1r16f were substituted with:
PNA_E_CR_V: TCAACCCT-TT CCC AAT ATT TAG GC-AGA GGC ATA CAA CGC C
PNA_I_CR_V: AA CAT GTA TCT GCG AA-CGA GTA GAA CAG TTG A TACTTATA

2.2. Corner Strand

t7r29f was substituted with:
PNA_7: CTA CAT TTT GAC GCT CAC GCT CAT GGA AAT AC TTT TACTTATATCAACCCT

2.3. Side

For the side position on the M13 scaffold the staple strand; t1r0g was left out.

2.4. Fluorescence Strands

Cy5-TAC TTA TAT CAA CCC T
AGG GTT GAT ATA AGT A-3IAbRQSp
